# Biomarkers of neonatal skin barrier adaptation reveal substantial differences compared to adult skin

**DOI:** 10.1038/s41390-020-1035-y

**Published:** 2020-06-29

**Authors:** Marty O. Visscher, Andrew N. Carr, Jason Winget, Thomas Huggins, Charles C. Bascom, Robert Isfort, Karen Lammers, Vivek Narendran

**Affiliations:** 1 Cincinnati Children’s Hospital Medical Center, Cincinnati, OH USA; 2 James L. Winkle College of Pharmacy, University of Cincinnati, Cincinnati, OH USA; 3 The Procter & Gamble Company, Cincinnati, OH USA

## Abstract

**Background:**

The objective of this study was to measure skin characteristics in premature (PT), late preterm (LPT), and full-term (FT) neonates compared with adults at two times (T1, T2).

**Methods:**

Skin samples of 61 neonates and 34 adults were analyzed for protein biomarkers, natural moisturizing factor (NMF), and biophysical parameters. Infant groups were: <34 weeks (PT), 34–<37 weeks (LPT), and ≥37 weeks (FT).

**Results:**

Forty proteins were differentially expressed in FT infant skin, 38 in LPT infant skin, and 12 in PT infant skin compared with adult skin at T1. At T2, 40 proteins were differentially expressed in FT infants, 38 in LPT infants, and 54 in PT infants compared with adults. All proteins were increased at both times, except TMG3, S100A7, and PEBP1, and decreased in PTs at T1. The proteins are involved in filaggrin processing, protease inhibition/enzyme regulation, and antimicrobial function. Eight proteins were decreased in PT skin compared with FT skin at T1. LPT and FT proteins were generally comparable at both times. Total NMF was lower in infants than adults at T1, but higher in infants at T2.

**Conclusions:**

Neonates respond to the physiological transitions at birth by upregulating processes that drive the production of lower pH of the skin and water-binding NMF components, prevent protease activity leading to desquamation, and increase the barrier antimicrobial properties.

**Impact:**

- Neonates respond to the transitions at birth by upregulating processes that drive the production of lower pH of the skin and NMF, prevent protease activity leading to desquamation, and increase the antimicrobial properties of the barrier.
- The neonatal epidermal barrier exhibits a markedly different array of protein biomarkers both shortly after birth and 2–3 months later, which are differentially expressed versus adults.
- The major biomarker-functional classes included filaggrin processing, protease inhibitor/enzyme regulators, antimicrobials, keratins, lipids, and cathepsins.
- The findings will guide improvement of infant skin care practices, particularly for the most premature infants with the ultimate goals mitigating nosocomial infection.

## Introduction

Newborn infants transition rapidly from a warm, wet, vernix-laden setting to a dry, cooler, environment at birth. Premature (PT) infants have an underdeveloped epidermal barrier with few cornified layers, increasing their risk for delayed skin development, permeability by noxious agents, and infection.^1–3^ The dermis is deficient in structural proteins, and the skin is more easily torn.^4^ Stratum corneum (SC) development after birth is rapid in very PT infant skin once exposed to a dry environment.^5–7^ Very PT infant SC is not fully competent, even at 1 month of life, with a significantly higher transepidermal water loss (TEWL) than full-term (FT) infants.^8^ The time to complete SC formation may be as long as 9 weeks postnatal age^5,8–10^ and longer for complete acid mantle development.^11^ At 23 weeks gestational age (GA), the SC is nearly absent with TEWL of ~75 g/m^2^/h.^12^ By week 26, TEWL is ~45 g/m^2^/h, corresponding to wounded skin.^1,2^ At 29 weeks of adjusted age, TEWL is ~17 g/m^2^/h, markedly higher than 5–6 g/m^2^/h for FTs. Very PT infants frequently exhibit abnormal desquamation after birth, indicating a hyperproliferative SC.

Significant differences in innate immune biomarkers, including structural proteins, were observed in PT infant skin versus FT neonatal and adult skin.^13^ Involucrin, albumin, proinflammatory cytokines IL-1β (interleukin-1β), IL-6, MCP-1 (monocyte chemoattractant protein-1), and IL-8 were significantly higher in infants ≤32 weeks of GA versus FT infants and adults. Both infant groups had significantly higher IL-1α and lower keratin^1,10,11^ and tumor necrosis factor-α than adults. Involucrin, higher in FTs than adults, and albumin levels were inversely related to GA.

While the skin changes rapidly after birth, the time course of development after birth to a fully functional, protective barrier is largely unknown. The overall, central study objective was to evaluate skin barrier development after birth in PT infant skin versus FT infant and adult skin, by determining the rate and time to functional integrity as a function of GA with proteomic analysis of biomarkers of skin barrier development and quantitative measures of TEWL, hydration, pH, dryness, and erythema. The present report focuses on the comparison of infant with adult skin.

## Methods

Infants and their parents were recruited from the Regional Center for Newborn Intensive Care (Level IV NICU) of Cincinnati Children’s Hospital Medical Center. The Institutional Review Board approved the research. Parents provided written informed consent. Infant exclusions were <24 weeks of GA, skin conditions, for example, ichthyosis and epidermolysis bullosa, and medical instability. Adult exclusions were active skin disease, for example, atopic dermatitis, scars, wounds, or damage. This trial was registered in ClinicalTrials.gov Identifier: NCT01619228.

### Skin surface samples and instrumental measures

Neonatal left and right lateral thigh/leg skin sites were examined at enrollment (day 1), days 4, 7, 11, and 14, weekly until discharge, and months 1, 3, 6, and 12 at outpatient visits. Adult volar forearms were evaluated once. Topical emollients were not applied to the test sites during the study. Infants were bathed once a week as per the NICU standard of care. Skin evaluations were made at least 8 h after bathing. Adult subjects refrained from emollient application on the volar forearms for 24 h prior to measurements. Skin surface samples were collected with 380-mm^2^ D-Squame® discs (CuDerm Corporation, Dallas, TX) from adult forearms (adjacent to instrumental sites) and one of five sites along the lower infant legs. The sites were first gently wiped with sterile water and soft medical gauze to remove potential contaminants from the skin surface. Following a standardized protocol, three sequential d’squames were applied with uniform pressure, left in place for 1 min, gently removed, and stored at −80 °C.^13^ Skin sites were assessed for visual erythema and dryness/scaling with standardized scales.^11,14–16^ TEWL (g/m^2^/h) was measured with a closed-chamber device (VapoMeter, Delfin Technologies, Ltd, Finland). Hydration was determined with the NOVA meter 9003 (NOVATechnology, MA). Skin pH was measured with a flat-electrode pH meter (Skincheck™, Hanna Instruments, UK).

### Proteomics sample preparation

The first two of the three d’squame tapes from each evaluation session were combined in the same tube and prepared for liquid chromatography with tandem mass spectrometry (LC-MS/MS) analysis by immersion in 1.5 mL of 50 mM ammonium bicarbonate and sonication for 30 min at ambient temperature. The resulting extract was dried to completion in a SpeedVac (Thermo Fisher Scientific, Waltham, MA). Dried protein was reconstituted in 100 μL of buffer containing 50 mM ammonium bicarbonate, 10 mM dithiothreitol, and a mixture of isotopically labeled peptide standards. Samples were incubated for 30 min at 56 °C. Iodoacetamide was then added to a final concentration of 25 mM, and then the samples were incubated for 30 min at ambient temperature in the dark. Next, 0.5 μg of Trypsin/LysC (Promega Corporation, Madison, WI) was added, and samples were digested for 4 h at 37 °C. Digestion was halted by addition of formic acid to a final concentration of 2%. Samples were dried to completion. Peptides were reconstituted in 40 μL of mobile phase A containing 0.1% formic acid in water. Ten microliters of the sample was injected and analyzed by LC-MS/MS.

### Proteomics strategy and targeted proteomics data collection

We employed targeted proteomics versus a shotgun, discovery approach. Target selection was based on over 30 human trials, including some shotgun analyses. The studies encompassed a wide age range and status, including normal, compromised (e.g., irritation and ultraviolet exposure), and diseased skin^17^ (data on file, Procter & Gamble). The targeted approach successfully detected distinct biology for the research questions in the present study. Multiple-reaction profiling, used in this study, provided greater precision than shotgun approaches.^18^ Targeted proteomics was shown to be the best approach for translation of proteomics data to the clinic.^19^ The throughput for shotgun analysis is typically 25-fold slower than targeted approaches. The faster throughput with the targeted approach allowed all individual samples to be analyzed without pooling responses across individual subjects. Analysis of many unique samples provides the strongest statistical power to detect quantitative biomarker differences.^20^ The top enriched gene ontology terms associated with the panel were: epidermal development, skin barrier establishment, keratinization, microbial defense, innate immunity, reactive oxygen species response, inflammation, and aging.

A pool was generated from all samples (including multiple timepoints per subject) and screened against the 158 targets in the previously defined multiple-reaction monitoring (MRM) panel. Seventy-three protein targets were selected (88 peptides and 352 transitions),^21^ and all individual samples were run against them (Supplementary Table S1 online). Samples were analyzed using an Agilent 6490 QQQ (Agilent Technologies, Fort Collins, CO) against a scheduled MRM method set to a 1000-ms cycle time with a 0.5-min retention time windows. The column dimensions were 2.1 × 150-mm^2^ C18, 1.8-μm particle size (Agilent Technologies, Fort Collins, CO), heated to 50 °C. The LC flow rate was 0.4 mL/min. Mobile phase A was 0.1% formic acid in water and mobile phase B was 0.1% formic acid in 1:9 water:acetonitrile. The LC gradient was from 97% A to 80.4% A from 0.67 to 5.67 min, and then to 73% A at 7.67 min, 50% A at 8.97 min, 10% A at 9.33 min, held at 10% A until 11 min, and finally to 97% A at 12 min. There was a 3-min post-run hold at 97% A to re-equilibrate the column.

### Natural moisturizing factor analysis

The d’squame extracts were analyzed for natural moisturizing factor (NMF) with LC/MS/MS and total protein by the bicinchoninic acid assay (Pierce, Thermo Fisher Scientific).^22^ Histidine, 2-pyrrolidone-5-acid (PCA), *cis*-urocanic acid (cisUCA), trans-UCA (transUCA), and proline were quantified, with total NMF as the sum, as previously described.^23^ NMF values were normalized to protein to account for differences in protein amount on the d’squame tapes.

### Proteomics data analysis

Raw data were imported into Skyline v. 4.3.0.19009 (University of Washington), and a mProphet (Biognosys, Inc., Boston, MA) model based on second-best peaks was trained to enable automatic peak selection. Peaks with a *q* value < 0.005 were selected, and some minor manual corrections were made. Feature-level peak areas were exported and processed further in R using MSstats v. 3.7.3 (Bioconductor) to perform normalization to protein and abundance estimation.

### Outcome data analysis

Infants were grouped by GA: <34 weeks GA (PT), 34–<37 weeks GA (late PT, LPT), and ≥37 weeks of GA (FT). All infant and adult outcomes were compared at both T1 and T2 using general linear models and post hoc assessments by Bonferroni (SPSS, SPSS Inc.) and significance at *p* < 0.05.

## Results

### Subjects for central study

Of 285 screened patients, 68 infants and 34 adults were enrolled for the central study (Supplementary Fig. S1 online). Four infants withdrew before any measurements were made. One infant died from complications unrelated to the study, after participation for several weeks and a period of medical instability.

### Subjects for the present report: infant–adult comparisons at two times

Sixty-one of the 64 infants had initial measurements made within 12 days after birth and were analyzed at T1 (Table 1). To examine the effect of time, data from assessments ~2–3 months later (T2) were compiled. Since the infants were enrolled for variable times based on length of stay, medical status, and ability to return for follow-up, the numbers of infants per group were smaller at T2 than T1. T2 was selected so that the PT, LPT, and FT groups were of comparable postconceptual ages (Table 1). Data from 34 adults (23 females and 11 males) represented mature, stable skin controls.

**Table 1:** Demographic characteristics.

	<34 weeks of GA (preterm, PT)	34–<37 weeks of GA (late preterm, LPT)	≥37 weeks of GA (full-term, FT)
Soon after birth, T1			
Number	11	18	32
Gestational age (weeks)	31.9 ± 0.3	35.3 ± 0.2	38.8 ± 0.2
Postconceptual age (days)	228 ± 2	255 ± 2	276 ± 1
Days from birth	5.3 ± 1.0	8.1 ± 0.8	4.4 ± 0.6
Gender (F/M)	4/7	9/9	15/17
Race (Caucasian/African American)	10/1	18/0	30/2
2–3 months later, T2			
Number	10	12	23
Gestational age (weeks)	32.0 ± 0.3	35.4 ± 0.3	39.1 ± 0.2
Postconceptual age (weeks)	324.7 ± 11.3	332.6 ± 10.3	337.6 ± 7.5
Days from birth	100.4 ± 11.8	84.8 ± 10.8	64.2 ± 7.8
Gender (F/M)	3/7	4/8	8/15
Race (Caucasian/African American)	9/1	12/0	23/0

The characteristics for subjects in the three infant groups soon after birth (T1) and 2–3 months later, and reported as mean ± SEM.

### Differential expression of protein biomarkers

#### Infants versus adults

**Fig. 1:**
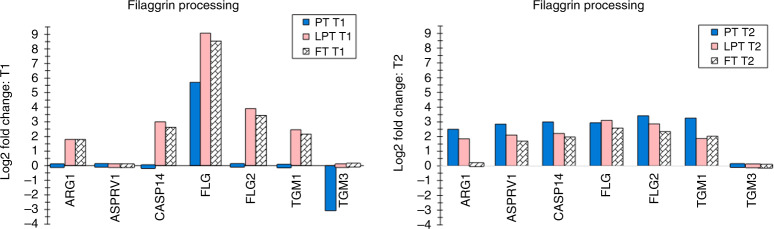
Filaggrin-processing proteins. Differentially expressed filaggrin-processing proteins (reported as log2 fold change) are shown for premature (PT), late premature (LPT), and full-term (FT) infants versus adults soon after birth (T1) and 2–3 months later (T2), when the mean postconceptional ages were comparable for the infant groups. The small bars indicate no (0 value) difference for the protein for the infant group versus adults. For example, ARG1, ASPRV1, CASP14, FLG2, and TGM1 were not differentially expressed for premature infants versus adults at T1 (premature infants).

**Fig. 2:**
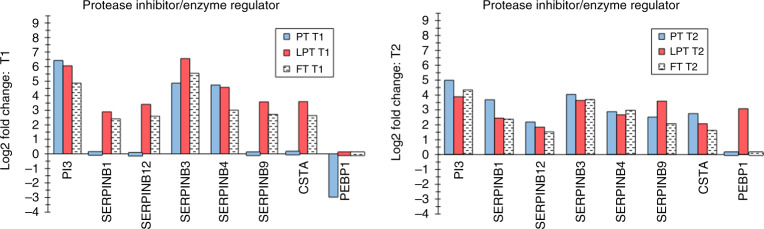
Protease inhibitor/enzyme regulating proteins. Differentially expressed protease inhibitor/enzyme-regulating proteins are shown for the three infant groups at T1 and T2.

**Fig. 3:**
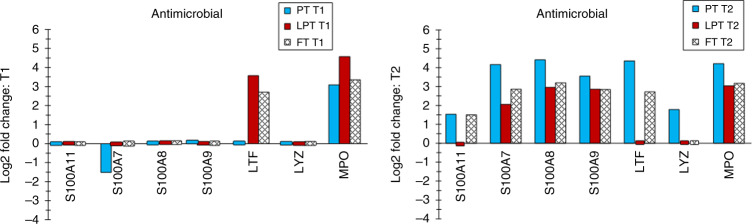
Antimicrobial proteins. Differentially expressed antimicrobial proteins are shown for PT, LPT, and FT infants compared with adults at T1 and T2.

**Fig. 4:**
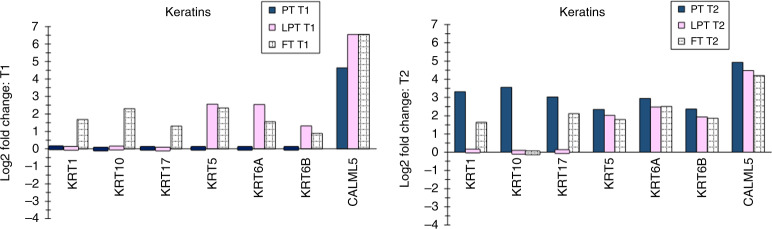
Keratin/structural proteins. Keratin/structural proteins that were differentially expressed for the three infant groups compared with adults are shown for T1 and T2.

**Fig. 5:**
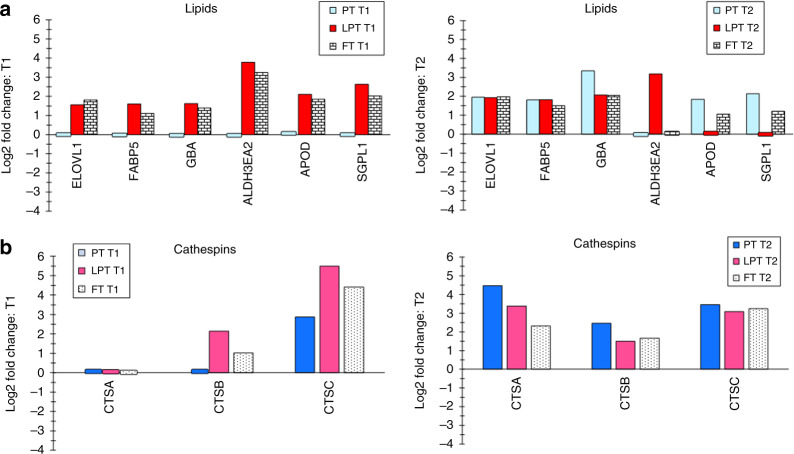
(a) Lipid processing proteins and (b) cathespin proteases. **a**, **b** Differentially expressed proteins involved with lipid processing (**a**) and the cathespin proteases (CTSA, CTSB, and CTSC) (**b**) are shown at T1 and T2 for the three infant groups.

Forty protein biomarkers were differentially expressed, that is, increased for FT infants versus adults at T1 and 46 were increased at T2 (*p* < 0.05). Thirty-eight biomarkers were differentially expressed for LPT infants versus adults at T1 and 46 were increased at T2 (*p* < 0.05). They are shown by functional classes: filaggrin processing (Fig. 1), protease inhibitor/enzyme regulators (Fig. 2), antimicrobials (Fig. 3), keratins/structural proteins (Fig. 4), lipid processing (Fig. 5a), and cathepsins (Fig. 5b) (*p* < 0.05). For PT infants, 12 and 54 proteins were differentially expressed at T1 and T2, respectively (Figs. 1–5). Comparing PT infants with adults at T1, nine biomarkers were increased, while three were decreased (Figs. 1–5). For PT infants at T2, 54 were increased compared with adults (Figs. 1–5). Supplementary Table S2 online provides the differentially expressed proteins for all three infant groups versus adults at both times, which are not represented in Figs. 1–5.

### Comparison of infant groups

Eight proteins were decreased in PTs compared to FTs (Table 2) with no differences between PTs and FTs at T2. There were no biomarker differences in LPT compared with FT at T1. For LPT versus FT infants at T2, seven proteins increased and five proteins decreased (Table 3).

**Table 2:** Differentially expressed proteins for premature infants versus full-term infants at T1.

Biomarker	Log2 fold change^a^	*p* Value
TMG3	−3.638	0.001154
FLG	−2.832	0.044382
CSTA	−2.697	0.041143
S100A8	−2.581	0.033581
ARG1	−2.533	0.033581
FGL2	−2.378	0.033581
CTSA	−1.604	0.033581
TTSA3	−0.409	0.033581

^a^Negative values indicate decrease for premature infants versus full-term infants.

**Table 3:** Differentially expressed proteins for late premature versus full-term infants at T2.

Biomarker	Log2 fold change^a^	*p* Value
FLG	6.498	3.75E−07
SERPINB3	2.860	0.000173
CALML5	2.340	0.044283
CTSC	2.255	0.013603
TF	2.175	0.00211
SERPINB12	1.885	0.034149
PI3	1.709	0.01907
DDAH2	−1.778	0.013603
LY6D	−1.988	0.00567
MDH2	−2.115	0.001034
SFN	−2.949	0.037004
S100A7	−3.457	3.75E−07

^a^Negative values indicate decreased protein for late premature infants versus full-term infants and positive values indicate increased protein for late premature infants versus full-term infants.

### Biomarker quantities

#### Infants versus parents

Biomarker quantities differed significantly for the infant groups versus adults at both T1 and T2 (*p* < 0.05). As expected, the infant–adult comparisons reaching significance paralleled those described above. For example, filaggrin-processing proteins FLG, FLG2, ARG1, and CASP14 were higher in FT versus adults at both times. TGM3 was higher in FT at T1, and ASPRV1 was higher at T2. FLG, FLG2, and CASP14 were higher in LPT versus adults at both times, while ASPRV1 was higher at T2. FLG was higher and TGM3 was lower in PT than adults at T1, and all except TGM3 were higher at T2. Supplementary Table S3 online shows the biomarker quantities and statistical comparisons for protease inhibitors/enzyme regulators, antimicrobial proteins, keratins, lipids, and cathespins for the infant groups versus adults at both times.

#### Filaggrin-processing products: NMF

Skin barrier function was assessed by quantifying the “products” of filaggrin processing, namely, NMF. Total NMF, PCA, histidine, proline, and histidine/total UCA were higher for adults than all infants at T1 (*p* < 0.05, Supplementary Table S4 online). CisUCA was not detected in PTs (Supplementary Table S4 online), but was lower in LPT and FT versus adults, as was cisUCA/total UCA. At T2, total NMF, PCA, proline, and transUCA were higher for all infants than adults (*p* < 0.05), with histidine higher for PT and LPT only. Infant cisUCA, histidine/totalUCA, and cisUCA/total UCA were lower than adults at T2 (*p* < 0.05).

### Biophysical outcomes

Protein amounts from the d’squames were higher for LPT and FT versus PT and adult samples at T1, indicating lower SC cohesion for LPT and FT groups (*p* < 0.05) (Table 4). At T2, protein was higher for all infants than adults (*p* < 0.05), signifying lower infant SC cohesion. At T1, TEWL was higher in PT versus FT (*p* < 0.05, Table 4). At T1, LPT and FT skin hydration was lower than adults (*p* < 0.05). Visual dryness was higher for PT and FT than adults at T1 and higher for FT than adults at T2 (*p* < 0.05). Skin pH was lower for LPT and FT than adults at T2 (*p* < 0.05). Erythema was higher for all infants at T1 (*p* < 0.05).

**Table 4:** Biophysical outcomes.

	<34 weeks of GA (PT)	34–<37 weeks of GA (LPT)	≥37 weeks of GA (FT)	Adults (Ad)	Statistics^a^
Soon after birth, T1					
TEWL (g/m^2^/h)	14.0 ± 1.6	10.4 ± 1.2	8.7 ± 0.9	10.0 ± 0.9	PT -S- versus FT
Hydration	106.2 ± 4.1	101.2 ± 3.2	93.8 ± 2.4	116.2 ± 2.3	LPT, FT -S- versus Ad
pH	6.05 ± 0.18	5.88 ± 0.14	6.09 ± 0.10	5.71 ± 0.10	*p* = 0.06, no pairwise differences
Erythema	1.1 ± 0.2	1.0 ± 0.2	0.8 ± 0.1	0.1 ± 0.1	PT, LPT, FT -S- versus Ad
Dryness	0.7 ± 0.2	0.7 ± 0.2	1.1 ± 0.1	0.0 ± 0.1	PT, LPT, FT -S- versus Ad
Protein (μg/mL)	16.0 ± 3.5	34.2 ± 2.8	35.8 ± 2.1	9.5 ± 2.1	LPT, FT -S- versus PT, Ad
Log10 Total NMF	1.87 ± 0.11	1.95 ± 0.09	1.67 ± 0.07	2.39 ± 0.06	PT, LPT, FT -S- versus Ad
2–3 months later, T2					
TEWL (g/m^2^/h)	11.8 ± 2.8	13.2 ± 2.3	15.9 ± 1.6	10.0 ± 0.9	FT -versus- Ad, *p* = 0.06
Hydration	113.2 ± 6.2	110.2 ± 5.1	110.8 ± 3.7	116.2 ± 2.3	-NS-
pH	5.44 ± 0.19	5.12 ± 0.16	5.23 ± 0.11	5.71 ± 0.10	LPT, FT -S- versus Ad
Erythema	0.3 ± 0.2	0.2 ± 0.2	0.4 ± 0.1	0.1 ± 0.1	-NS-
Dryness	0.5 ± 0.2	0.4 ± 0.1	0.5 ± 0.1	0.0 ± 0.1	FT -S- versus Ad
Protein (μg/mL)	21.2 ± 2.1	18.8 ± 1.9	16.2 ± 1.4	9.5 ± 2.1	PT, LPT, FT -S- versus Ad
Log10 Total NMF	2.81 ± 0.07	2.72 ± 0.06	2.72 ± 0.04	2.39 ± 0.06	PT, LPT, FT -S- versus Ad

^a^Indicated here are the post hoc comparisons (Bonferroni) with *p* < 0.05 from general linear model analyses of the four groups where the model *F*-statistic is significant at *p* < 0.05.

*-S-* significant pairwise comparisons, *-NS-* not significant, *PT* infants <34 weeks GA, *LPT* 34–<37 weeks GA, *FT* full-term (≥37 weeks GA).

The biophysical measurement results for the three infant groups and adults are shown provided for soon after birth (T1) and 2–3 months later (T2).

## Discussion

We aimed to assess skin barrier adaptation and functional integrity in PT, LPT versus FT infants using targeted proteomic analysis of skin biomarkers and clinical measures. Neonates responded to the transitions at birth to upregulate processes that drive skin pH lowering and production of water-binding NMF, prevent protease-based desquamation, and increase the epidermal barrier antimicrobial function. A markedly different array of protein biomarkers was observed shortly after birth and 2–3 months later versus stable adult skin. The barrier changed over time with varying patterns, depending upon infant GA. Specific proteins were decreased in PT infants <34 weeks of GA versus FT infants, then increased, and finally became indistinguishable from FT 2–3 months later. LPT and FT biomarker profiles did not differ at T1, indicating similar barrier status. The infant groups had 39 biomarkers in common at T2, corresponding to 72% of the increased proteins for PT and 78% for LPT and FT infants. The number of differentially expressed proteins increased for PT infants versus adults (from 12 to 54), suggesting substantial adaptive changes over time.

The results confirm that neonatal and adult skin differs. They suggest that neonatal skin is designed to provide innate immunity and protection from environmental effects and substantiate rapid barrier continued development after birth. Many of the increased proteins involve late differentiation, cornification, and filaggrin processing. Differences in filaggrin-processing proteins between infants and adults lead to higher NMF levels for infants over time, regardless of GA. PI3 (elafin), CSTA, and the SERPINB proteins were increased in infants. They inhibit proteases, regulate enzymes, have antimicrobial properties, and process filaggrin to generate NMF and decrease skin pH. To our knowledge, this is the first report of the detailed early-life epidermal barrier changes.

Nine proteins were increased in all infant groups versus adults at T1, namely FLG, SERPINB3, SERPINB4, PI3, MPO, CALML5, CTSC, ALB, and TF (Figs. 1, 2, 3, and 5b, Supplementary Table S2 online), implying their importance in neonatal skin adaptation. MPO (antimicrobial, Fig. 3) is a neutrophil marker and may increase due to inflammation.^24^ Calcium-dependent CALML5 (Fig. 4) controls terminal epidermal differentiation^25^ and participates in barrier restoration in atopic dermatitis.^26^ High CALML5 levels were reported as necessary for binding of TMG3 in cornified envelope formation in psoriasis.^27^ The cysteine enzyme CTSC (Fig. 5b) stimulates immune cell serine proteases and assists in tissue structural organization and keratin protein processing.^28^ Suprabasal epidermal ALB occurred via diffusion through the basement membrane.^29^ ALB was found in epidermal keratinocytes and suction blisters, confirming synthesis in the epidermis versus transport from serum.^30^ ALB levels were significantly higher in atopic lesions versus uninvolved atopic skin and nonatopic controls.^31^ TF was implicated in barrier defense and inflammatory responses.^32^

Three proteins were decreased, but only in PT infants <34 weeks of GA (Table 2). PEBP1, a serine protease inhibitor of chymotrypsin, has been implicated in oxidative stress^32^ and regulation of epidermal differentiation.^33^ Deficiencies in mechanisms to promote skin adaptation and to manage the high oxidative stress are problematic for the PT infants.

### Filaggrin processing

Biomarkers from two of four epidermal differentiation complex protein classes^34^ were increased in infants, that is, S100 fused proteins and S100 proteins. Shortly after birth, filaggrin-processing proteins, FLG, FLG2, TGM3, CASP14, and ARG1, were increased in FT. Only FLG was increased in PT (Fig. 1). After 2–3 months, the proteins were higher in FT and, except TGM3, were increased in PT versus adults (Fig. 1). BHLM and PADI1 were not differentially expressed.

Filaggrin proteolysis was inhibited at relative humidities (RHs) >95% and <~75% in animals.^35^ NMF production took longer when animals transitioned from 80 to 10% RH than from 40–70 to 10%.^36^ Neonates transfer from 100 to ~40–50% RH at birth, although extremely PT infants are typically housed at 75–80% RH to reduce water loss.

PT infants <34 weeks of GA had reduced FLG, FLG2, TMG3, and ARG1 versus FT shortly after birth (Table 2). Filaggrin was reduced in atopic dermatitis, under stressful conditions, and with exposure to irritants and certain cytokines.^37^ In this study, higher infant FLG was associated with lower NMF soon after birth (Table 4), suggesting that FLG proteolysis was limited. After 2–3 months, FLG remained higher in infants than adults (Table 4), but total NMF, PCA, histidine, and proline were significantly higher in infants than adults (Supplementary Table S4 online). Filaggrin proteolysis to NMF contributes to acid mantle formation.^38^ Increased NMF was associated with lower pH 2–3 months later in FT versus adults (Table 4).

FLG2 was decreased in PT versus FT infants shortly after birth (Table 2). FLG2 expression occurred in terminally differentiated keratinocytes, and was reduced in psoriasis, an inflammatory disease.^39^ FLG2 was significantly lower than normal in the essential fatty acid-deficiency model of skin barrier compromise. Regulation of FLG2 activity may occur differently than FLG.^40^ Cell–cell cohesion was lower in keratinocytes from subjects with reduced FLG2 levels.^41^

Transglutaminase 3 was found in fetal epidermis at 23 weeks of GA,^42^ consistent with our detection in PT, although at reduced levels. FLG and FLG2 were decreased in tape-stripped skin,^43^ similar to our lower levels for PT versus FT. However, transglutaminases 1, 3, and 5 were upregulated, in contrast to our findings. ASPRV1 is important for filaggrin processing and SC hydration.^44^ We found ASPRV1 to be increased in PT and FT versus adults 2–3 months after birth (Fig. 1); at the same time NMF was also found to be higher when compared with that in adults (Table 4).

While NMF was lower for infants versus adults soon after birth, NMF increased substantially over 2–3 months to higher than adult levels (Table 4, Supplementary Table S4 online). The NMF increase is consistent with previous reports of low values at birth in FT infants (<48-h old), increasing from 48 h to 4 weeks and further increasing over 1–11 months.^45^ NMF participates in acid mantle formation.^46^ When exposed to UCA plus PCA, *Staphylococcus aureus* growth was reduced and cell density lowered.^47^ Both effects are important for neonates who rely upon innate immunity for protection.

### Protease inhibitors/enzyme regulators

Protease inhibitors PI3 (elafin), SERPINB3, and SERPINB4 (Fig. 2), were increased in infants at both times, suggesting an important role in SC adaptation. Elafin inhibits human leukocyte elastase, appearing in the granular and upper spinosum by gestational weeks 28–29.^48^ It inhibited kallikrein (KLK5 and KLK7) proteolytic activity^49^ and desquamation,^50^ and participated in corneocyte envelope formation at terminal differentiation.^51^ Reduced desquamation would be protective for PT infants, that is, until the SC is sufficiently functional. Elafin in eccrine ducts and hair follicles was bactericidal against *Pseudomonas aeruginosa* and *S. aureus*.^52^ SC antimicrobial functionality at birth would benefit neonates at birth. SERPINB3 inhibited cysteine proteases, and SERPINB4 influenced chymotrypsin enzymes, functions that may extend opportunistic bacteria.^53^ SERPINB3 was upregulated in barrier compromise, for example, atopic dermatitis, psoriasis, and irritant exposure,^54^ suggesting provision of immunity.^53^

### Antimicrobials

Several antimicrobial proteins, including S100 proteins and MPO, were increased in infants, generally not until the later time (T2) (Fig. 3). S100 protein increases may arise from exposure to cytokines and other stressors.^55,56^ S100A7, S100A8, and S100A9 increased filaggrin production and decreased proliferation,^57^ suggesting performance of specific tasks in response to insult.

Interestingly, specific precursors of cornified envelope formation, including loricrin, involucrin, and desmoglein-3, were not differentially expressed, implying their presence in all infants. TGM3 was implicated in cross-linking with loricrin at lysine and glutamine residues during envelope formation.^58^ Elafin and the cystatins (e.g., CSTA) regulate proteases in envelope maturation.^58^ Elafin and CSTA were increased in infants, and CSTA decreased in PT versus FT, perhaps indicating that the neonatal barrier is in late-stage envelope formation.

Vernix caseosa gradually covers the skin surface during the last trimester. FT samples contained proteins with innate immune properties, including S100A7, S100A8, S100A9, CTSA, CASP14, ARG1, TF, KRT10, TXN, ALB, HIST1H4A, HIST1H3A, LYZ, SERPINB3, and SERPINB4.^59^ Some were increased in infants at T1. The antimicrobials S100A7, S100A8, and S100A9 were not, but their levels had increased by T2. This finding highlights the importance of the innate immune function of vernix at birth. The infants <34 weeks of GA did not likely have significant vernix at birth.^60^ Furthermore, S100A8 was decreased in this group versus FT infants.

Some specific features were noteworthy, as they emphasize the utility of the findings and potential limitations. While we did not employ discovery protein analysis, our targeted protein panel was based on previous studies identifying key skin proteins that were detectable from d’squame tapes.^17^ We used a set of well-characterized markers of epidermal development and homeostasis to help understand the state of the SC, rather than conducting a comprehensive proteomic characterization of infant skin. In addition, SC thickness varies with GA and time from birth^61^ and varied across subjects. Therefore, biomarker and NMF values were normalized to the material (protein) removed from the skin surface with tapes. However, the protein amounts varied where quantities were higher for LPT and FT versus PT and adults at T1. The skin surface collection method does not sample deeply, but permitted characterization of the outer infant SC at various developmental times. About 20–30 tapes are required to remove the entire SC.^62^ We collected only three sequential tapes to avoid any discomfort or injury. We saw no evidence of reaching the viable epidermis, for example, irritation and glistening. McAleer et al.^45^ addressed the question of SC thickness and NMF by collecting eight tape strips from FT infants and found no differences in NMF levels. We were unable to conduct a similar experiment due to limitations in tolerance and the potential for injury, particularly in the PT group.

The study did not include infants ≤29 weeks o GA. We did not determine when PT protein biomarkers reached comparable expression to FT infants. Additional research on extremely PT infants is warranted to fully characterize skin barrier adaptation. We anticipate that future reports will compare biomarkers for the infant groups over time by functional class. We sampled from different body sites on infants (legs) versus adults (arms) and, therefore, did not account for potential site differences. The lower legs of hospitalized infants are generally free of lines, dressings, and tapes that would alter the SC, in contrast to their arms, chest, and face. The larger area allowed sampling of different sites to avoid resampling the same area. Adult volar forearms are largely free of hair and are not subject to damage from shaving.

The collective findings provide fundamental knowledge on PT and FT infant skin development, and reveal important differences between adapting infant and adult skin. The PT infant skin phenotype appears to be resistant to desquamation and ensures sufficient antimicrobial defense. Upregulation of multiple filaggrin-processing proteins and the production of NMF at levels higher than adults suggest that acid mantle formation, antimicrobial capability, and protection against early desquamation are critical for PT and FT infant survival. The results will guide improvement of infant skin care practices, particularly for the most PT infants with the ultimate goals mitigating nosocomial infection.

## Supplementary information

Supplementary tableS1

Supplementary tableS2

Supplementary tableS3

Supplementary tableS4

Supplementary figure
