# Correction: Biomarkers of neonatal skin barrier adaptation reveal substantial differences compared to adult skin

**DOI:** 10.1038/s41390-020-01147-1

**Published:** 2020-09-14

**Authors:** Marty O. Visscher, Andrew N. Carr, Jason Winget, Thomas Huggins, Charles C. Bascom, Robert Isfort, Karen Lammers, Vivek Narendran

**Affiliations:** 1 Cincinnati Children’s Hospital Medical Center, Cincinnati, OH USA; 2 James L. Winkle College of Pharmacy, University of Cincinnati, Cincinnati, OH USA; 3 The Procter & Gamble Company, Cincinnati, OH USA

Correction to: *Pediatric Research* 10.1038/s41390-020-1035-y, published online 29 June 2020

This article was originally published without open access, but has now been made available under a [CC BY 4.0] license. The PDF and HTML versions of the paper have been modified accordingly.

