# Supplementary material for: Biomarkers of neonatal skin barrier adaptation reveal substantial differences compared to adult skin
Source: Pediatr Res. 2020 Jun 29;89(5):1208–15. doi: 10.1038/s41390-020-1035-y (PMC8119241; doi:10.1038/s41390-020-1035-y)
Supplement: Supplementary file 1 — Supplementary tableS1 [file 41390_2020_1035_MOESM1_ESM.docx]

**Supplementary Table S1**. Proteomics Target List. The list of targeted proteins and peptides is shown in the table.

| **Accession** | **GeneID** | **Peptide.Modified.Sequence** |
| --- | --- | --- |
| O15173 | PGRMC2 | FYGPAGPYGIFAGR |
| O75367 | H2AFY | GVTIASGGVLPNIHPELLAK |
| O95470 | SGPL1 | ALPSQGLSSSAVLEK |
| O95865 | DDAH2 | GGGDLPNSQEALQK |
| O95865 | DDAH2 | LSDVTLVPVSC[+57]SELEK |
| P01040 | CSTA | TQVVAGTNYYIK |
| P02538 | KRT6A | GMQDLVEDFK |
| P02538 | KRT6A | SGFSSVSVSR |
| P02768 | ALB | SLHTLFGDK |
| P02787 | TF | DGAGDVAFVK |
| P02788 | LTF | GGSFQLNELQGLK |
| P04062 | GBA | FIPEGSQR |
| P04062 | GBA | PVSLLASPWTSPTWLK |
| P04179 | SOD2 | GDVTAQIALQPALK |
| P04259 | KRT6B | ATGGGLSSVGGGSSTIK |
| P04259 | KRT6B | QLDNIVGER |
| P04264 | KRT1 | SLDLDSIIAEVK |
| P04406 | GAPDH | LVINGNPITIFQER |
| P05089 | ARG1 | VMEETLSYLLGR |
| P05090 | APOD | NILTSNNIDVK |
| P05109 | S100A8 | GNFHAVYR |
| P05109 | S100A8 | LLETEC[+57]PQYIR |
| P05164 | MPO | IANVFTNAFR |
| P05164 | MPO | VVLEGGIDPILR |
| P06702 | S100A9 | VIEHIMEDLDTNADK |
| P07858 | CTSB | GQDHC[+57]GIESEVVAGIPR |
| P08779 | KRT16 | APSTYGGGLSVSSR |
| P10599 | TXN | TAFQEALDAAGDK |
| P10619 | CTSA | EFSHIAFLTIK |
| P11142 | HSPA8 | DAGTIAGLNVLR |
| P13645 | KRT10 | ALEESNYELEGK |
| P13647 | KRT5 | SFSTASAITPSVSR |
| P16870 | CPE | DLQGNPIANATISVEGIDHDVTSAK |
| P19957 | PI3 | GPVSTKPGSC[+57]PIILIR |
| P20930 | FLG | HGSYGSADYDYGESGFR |
| P22735 | TGM1 | IYYGTEAQIGER |
| P29508 | SERPINB3 | FYQTSVESVDFANAPEESR |
| P29508 | SERPINB3 | VLHFDQVTENTTGK |
| P30086 | PEBP1 | NRPTSISWDGLDSGK |
| P30740 | SERPINB1 | TYNFLPEFLVSTQK |
| P31151 | S100A7 | GTNYLADVFEK |
| P31350 | RRM2 | VPLAPITDPQQLQLSPLK |
| P31944 | CASP14 | DPTAEQFQEELEK |
| P31947 | SFN | YLAEVATGDDK |
| P31949 | S100A11 | DGYNYTLSK |
| P35908 | KRT2 | VDLLNQEIEFLK |
| P40926 | MDH2 | IFGVTTLDIVR |
| P40926 | MDH2 | VAVLGASGGIGQPLSLLLK |
| P48594 | SERPINB4 | VLHFDQVTENTTEK |
| P50453 | SERPINB9 | HLGIVDAFQQGK |
| P51648 | ALDH3A2 | YIAPTVLTDVDPK |
| P53634 | CTSC | NVHGINFVSPVR |
| P56537 | EIF6 | HGLLVPNNTTDQELQHIR |
| P59665 | DEFA1 | IPAC[+57]IAGER |
| P61626 | LYZ | LGMDGYR |
| P61626 | LYZ | STDYGIFQINSR |
| P62805 | HIST1H4A | ISGLIYEETR |
| P68431 | HIST1H3A | STELLIR |
| Q01469 | FABP5 | ELGVGIALR |
| Q04695 | KRT17 | ALEEANTELEVK |
| Q08188 | TGM3 | SQGVFQC[+57]GPASVIGVR |
| Q13113 | PDZK1IP1 | YSSMAASFR |
| Q13630 | TSTA3 | DADLTDTAQTR |
| Q13630 | TSTA3 | ILVTGGSGLVGK |
| Q14206 | RCAN2 | IELHETQFR |
| Q14210 | LY6D | HSVVC[+57]PASSR |
| Q53RT3 | ASPRV1 | ILGVWDTAVSLGK |
| Q5D862 | FLG2 | SVVTVIDVFYK |
| Q5T9L3 | WLS | ITMMSRPPVLLEK |
| Q6P4A8 | PLBD1 | TTGWGILEIR |
| Q92561 | PHYHIP | VTHYFIDLNK |
| Q96P63 | SERPINB12 | ADLTGISPSPNLYLSK |
| Q99731 | CCL19 | NFHYLLIK |
| Q9BQ50 | TREX2 | SSLENPEHDESGALVLPR |
| Q9BW60 | ELOVL1 | VAWLFLFSK |
| Q9NZT1 | CALML5 | AGLEDLQVAFR |
