# Supplementary material for: Biomarkers of neonatal skin barrier adaptation reveal substantial differences compared to adult skin
Source: Pediatr Res. 2020 Jun 29;89(5):1208–15. doi: 10.1038/s41390-020-1035-y (PMC8119241; doi:10.1038/s41390-020-1035-y)
Supplement: Supplementary file 2 — Supplementary tableS2 [file 41390_2020_1035_MOESM2_ESM.docx]

**Supplementary Table S2**. The levels of differentially expressed proteins, not represented in Figure 1, are provided for all three infant groups versus adults at both times.

**Time 1 Late**

**Premature Full-Term**

|  | LPT log2FCh | adj pvalue |  |  | FT Log2Fch | adj pvalue |  |  |  |  |
| --- | --- | --- | --- | --- | --- | --- | --- | --- | --- | --- |
| ALB | 3.407 | 3.43E-06 |  | ALB | 3.515 | 3.12E-08 |  |  |  |  |
| H2AFY | 3.830 | 5.37E-04 |  | H2AFY | 4.119 | 1.42E-05 |  |  |  |  |
| HIST1H3A | 2.571 | 5.82E-03 |  | HIST1H3A | 2.351 | 2.56E-03 |  |  |  |  |
| HIST1H4A | 3.071 | 1.45E-03 |  | HSPA8 | 1.740 | 2.56E-03 |  |  |  |  |
| HSPA8 | 2.776 | 1.70E-04 |  | RRM2 | 2.145 | 4.30E-04 |  |  |  |  |
| RRM2 | 2.589 | 5.37E-04 |  | SOD2 | 1.325 | 1.82E-02 |  |  |  |  |
| SOD2 | 2.113 | 2.35E-03 |  | TF | 2.474 | 7.41E-07 |  |  |  |  |
| TF | 2.961 | 1.68E-06 |  | TGM1 | 2.156 | 7.77E-04 |  |  |  |  |
| TGM1 | 2.458 | 1.59E-03 |  | TREX2 | 1.947 | 1.50E-02 |  |  |  |  |
| TSTA3 | 0.432 | 7.16E-04 |  | TSTA3 | 0.277 | 6.65E-03 |  |  |  |  |
| TXN | 2.430 | 9.20E-03 |  |  |  |  |  |  |  |  |
| **Time 2** | **Late Premature** |  |  |  | **Full-Term** |  |  |  | **Premature** |  |
|  | LPT log2FCh | adj pvalue |  |  | FT Log2FCh | adj pvalue |  |  | PT Log2FCh | adj pvalue |
| ALB | 2.355 | 1.77E-03 |  | ALB | 2.626 | 5.71E-05 |  | ALB | 3.090 | 2.38E-04 |
| CCL19 | 0.913 | 1.35E-02 |  | DDAH2 | 1.509 | 3.02E-03 |  | CCL19 | 1.190 | 2.46E-03 |
| CPE | 3.800 | 1.46E-02 |  | EIF6 | 1.809 | 3.30E-03 |  | CPE | 4.450 | 5.71E-03 |
| DDAH2 | 2.401 | 5.18E-04 |  | H2AFY | 4.705 | 1.34E-05 |  | DDAH2 | 2.033 | 2.38E-03 |
| EIF6 | 2.151 | 6.22E-03 |  | HIST1H3A | 3.444 | 1.15E-04 |  | EIF6 | 3.018 | 4.09E-04 |
| H2AFY | 4.581 | 5.18E-04 |  | HIST1H4A | 3.289 | 2.48E-04 |  | GAPDH | 3.072 | 2.46E-03 |
| HIST1H3A | 3.712 | 9.20E-04 |  | HSPA8 | 2.163 | 8.78E-04 |  | H2AFY | 5.413 | 9.18E-05 |
| HIST1H4A | 3.239 | 3.02E-03 |  | LY6D | 1.768 | 1.20E-03 |  | HIST1H3A | 4.707 | 9.18E-05 |
| HSPA8 | 2.099 | 7.48E-03 |  | MDH2 | 1.890 | 1.15E-04 |  | HIST1H4A | 4.458 | 1.94E-04 |
| LY6D | 1.740 | 3.37E-03 |  | SFN | 3.244 | 1.92E-03 |  | HSPA8 | 2.969 | 4.83E-04 |
| MDH2 | 2.407 | 1.96E-04 |  | SOD2 | 2.702 | 5.84E-05 |  | LY6D | 2.011 | 1.94E-03 |
| RRM2 | 1.727 | 2.61E-02 |  | TGM1 | 2.012 | 3.29E-03 |  | MDH2 | 2.714 | 5.99E-05 |
| SFN | 2.514 | 3.14E-02 |  | TSTA3 | 0.349 | 1.92E-03 |  | PDZK1IP1 | 1.631 | 4.52E-02 |
| SOD2 | 3.254 | 1.96E-04 |  |  |  |  |  | RRM2 | 2.382 | 5.89E-03 |
| TGM1 | 1.861 | 2.53E-02 |  |  |  |  |  | SFN | 3.387 | 6.61E-03 |
| TREX2 | 2.320 | 2.53E-02 |  |  |  |  |  | SOD2 | 3.629 | 6.41E-05 |
| TXN | 2.503 | 1.35E-02 |  |  |  |  |  | TGM1 | 3.261 | 4.83E-04 |
|  |  |  |  |  |  |  |  | TSTA3 | 0.450 | 2.32E-03 |
|  |  |  |  |  |  |  |  | TXN | 2.512 | 1.54E-02 |
