# Supplementary material for: Biomarkers of neonatal skin barrier adaptation reveal substantial differences compared to adult skin
Source: Pediatr Res. 2020 Jun 29;89(5):1208–15. doi: 10.1038/s41390-020-1035-y (PMC8119241; doi:10.1038/s41390-020-1035-y)
Supplement: Supplementary file 3 — Supplementary tableS3 [file 41390_2020_1035_MOESM3_ESM.docx]

**Supplementary Table S3**. Biomarker Quantities (log values). * Indicates statistically significant difference versus adult values at p < 0.05.

Filaggrin Processing

|  | PT T1 | LPT T1 | FT T1 | Adult | PT T2 | LPT T2 | FT T2 | Adult |
| --- | --- | --- | --- | --- | --- | --- | --- | --- |
| ARG1 | 12.0 ± 0.6 | 14.1 ± 0.5 | 14.4 ± 0.4* | 12.7 ± 0.4 | 15.3 ± 0.8* | 14.6 ± 0.7 | 13.7 ± 0.5 | 12.7 ± 0.4 |
| ASPRV1 | 11.8 ± 0.5 | 13.0 ± 0.4 | 13.0 ± 0.3 | 12.2 ± 0.3 | 15.2 ± 0.7* | 14.4 ± 0.6* | 14.0* ± 0.4 | 12.2 ± 0.3 |
| CASP14 | 10.8 ± 0.7 | 13.0 ± 0.5* | 13.0 ± 0.4* | 10.4 ± 0.4 | 13.4 ± 0.6* | 12.6 ± 0.6* | 12.4 ± 0.4* | 10.4 ± 0.4 |
| FLG | 14.0 ± 0.9* | 17.1 ± 0.7* | 16.8 ± 0.5* | 8.3 ± 0.5 | 11.3 ± 0.9* | 11.3 ± 0.8* | 10.9 ± 0.6* | 8.3 ± 0.5 |
| FLG2 | 19.0 ± 0.6 | 21.7 ± 0.5* | 21.4 ± 0.4* | 18.0 ± 0.3 | 21.4 ± 0.7* | 20.8 ± 0.7* | 20.3 ± 0.5* | 18.0 ± 0.3 |
| TGM3 | 9.8 ± 0.7* | 12.8 ± 0.5 | 13.5± 0.4 | 13.2 ± 0.4 | 14.0 ± 0.7 | 13.1 ± 0.6 | 13.8 ± 0.5 | 13.2 ± 0.4 |

Protease Inhibitor/Enzyme Regulator

|  | PT T1 | LPT T1 | FT T1 | Adult | PT T2 | LPT T2 | FT T2 | Adult |
| --- | --- | --- | --- | --- | --- | --- | --- | --- |
| PI3 | 16.4 ±0.5* | 15.7 ± 0.4* | 14.8 ± 0.3* | 9.9 ± 0.3 | 14.9 ± 0.5* | 13.8 ± 0.5* | 14.3 ± 0.4* | 9.9 ± 0.3 |
| SERPINB1 | 10.0 ± 0.6 | 12.2 ± 0.5* | 11.7 ± 0.4* | 9.3 ± 0.3 | 13.0 ± 0.8* | 11.7 ± 0.7* | 11.7 ± 0.5* | 9.3 ± 0.3 |
| SERPINB12 | 15.7 ± 0.5 | 18.2 ± 0.4* | 17.6 ± 0.3* | 15.0 ± 0.3 | 17.3 ± 0.6* | 16.9 ± 0.6* | 16.6 ± 0.4* | 15.0 ± 0.3 |
| SERPINB3 | 13.5 ± 0.5* | 15.0 ± 0.4* | 14.1 ± 0.3* | 8.6 ± 0.3 | 12.8 ± 0.5* | 12.2 ± 0.5* | 12.3 ± 0.4* | 8.6 ± 0.3 |
| SERPINB4 | 12.2 ± 0.6* | 11.8 ± 0.4* | 10.4 ± 0.3* | 7.4 ± 0.3 | 10.5 ± 0.7* | 10.1 ± 0.7* | 10.4 ± 0.5* | 7.4 ± 0.4 |
| SERPINB9 | 8.6 ± 0.6 | 10.9 ± 0.5* | 10.1 ± 0.4* | 7.8 ± 0.4 | 9.7 ± 0.7 | 11.1 ± 0.6 | 9.2 ± 0.4* | 7.8 ± 0.4 |
| CSTA | 17.7 ± 0.4 | 19.1 ± 0.3* | 19.2 ± 0.2* | 16.6 ± 0.2 | 19.4 ± 0.5* | 18.7 ± 0.4* | 18.2 ± 0.3* | 16.6 ± 0.2 |
| PEBP1 | 5.7 ± 0.9* | 8.1 ± 0.7 | 7.2 ± 0.6 | 8.7 ± 0.5 | 11.0 ± 0.9 | 11.8 ± 0.8* | 9.6 ± 0.6 | 8.7 ± 0.5 |

Antimicrobials

|  | PT T1 | LPT T1 | FT T1 | Adult | PT T2 | LPT T2 | FT T2 | Adult |
| --- | --- | --- | --- | --- | --- | --- | --- | --- |
| S100A11 | 9.1 ± 0.5 | 9.9 ± 0.4 | 9.7 ± 0.3 | 9.4 ± 0.3 | 10.9 ± 0.6 | 10.4 ± 0.5 | 10.9 ± 0.4* | 9.4 ± 0.3 |
| S100A7 | 12.3 ± 0.6 | 14.2 ± 0.5 | 13.4 ± 0.4 | 13.8 ± 0.4 | 18.0 ± 0.5* | 15.9 ± 0.5* | 16.7 ± 0.4* | 13.8 ± 0.3 |
| S100A8 | 6.4 ± 0.7 | 9.2 ± 0.6 | 9.0 ± 0.4 | 8.3 ± 0.4 | 12.4 ± 0.6* | 11.0 ± 0.6* | 11.2 ± 0.4* | 8.3 ± 0.4 |
| S100A9 | 7.6 ± 1.1 | 9.6 ± 0.8* | 9.2 ± 0.6 | 6.7 ± 0.7 | 10.9 ± 1.1* | 10.4 ± 1.0* | 10.4 ± 0.7* | 6.7 ± 0.7 |
| LTF | 7.5 ± 1.2 | 10.4 ± 0.9* | 9.3 ± 0.7* | 5.5 ± 0.7 | 10.4 ± 1.2* | 9.2 ± 1.1* | 9.5 ± 0.8* | 5.5 ± 0.7 |
| LYZ | 12.6 ± 0.5 | 14.2 ± 0.4 | 14.0 ± 0.3 | 13.2 ± 0.3 | 14.9 ± 0.6 | 14.7 ± 0.6 | 14.3 ± 0.4 | 13.2 ± 0.3 |
| MPO | 9.0 ± 0.7* | 10.4 ± 0.5* | 9.2 ± 0.4* | 6.1 ± 0.4 | 10.0 ± 0.7* | 8.9 ± 0.6* | 9.0 ± 0.4* | 6.1 ± 0.4 |

Keratins

|  | PT T1 | LPT T1 | FT T1 | Adult | PT T2 | LPT T2 | FT T2 | Adult |
| --- | --- | --- | --- | --- | --- | --- | --- | --- |
| KRT1 | 21.0 ± 0.7 | 22.4 ± 0.5 | 22.4 ± 0.4* | 20.8 ± 0.4 | 24.1 ± 0.8* | 22.2 ± 0.7 | 22.4 ± 0.5 | 20.8 ± 0.4 |
| KRT10 | 18.6 ± 1.1 | 19.5 ± 0.9 | 19.8 ± 0.6 | 17.6 ± 0.6 | 20.2 ± 1.2 | 18.7 ± 1.1 | 19.4 ± 0.8 | 17.6 ± 0.6 |
| KRT17 | 9.4 ± 0.7 | 11.2 ± 0.5 | 11.0 ± 0.4 | 9.7 ± 0.4 | 12.9 ± 0.8* | 11.4 ± 0.7 | 11.9 ± 0.5* | 9.7 ± 0.4 |
| KRT5 | 15.5 ± 0.6 | 16.8 ± 0.4* | 16.7 ± 0.3* | 14.4 ± 0.3 | 16.9 ± 0.7* | 16.4 ± 0.6 | 16.2 ± 0.6* | 14.4 ± 0.3 |
| KRT6A | 9.6 ± 0.7 | 10.8 ± 0.6* | 10.0 ± 0.4* | 8.4 ± 0.4 | 11.6 ± 0.8* | 11.1 ± 0.8* | 11.1 ± 0.5* | 8.4 ± 0.4 |
| KRT6B | 9.1 ± 0.5 | 10.8 ± 0.4 | 10.6 ± 0.3 | 9.7 ± 0.3 | 12.1 ± 0.4* | 11.7 ± 0.4* | 11.5 ± 0.3* | 9.7 ± 0.3 |
| CALML5 | 13.2 ± 0.8* | 15.1 ± 0.6* | 15.1 ± 0.5* | 8.6 ± 0.5 | 13.5 ± 0.7* | 13.1 ± 0.7* | 12.8 ± 0.5* | 8.6 ± 0.5 |

Lipids

|  | PT T1 | LPT T1 | FT T1 | Adult | PT T2 | LPT T2 | FT T2 | Adult |
| --- | --- | --- | --- | --- | --- | --- | --- | --- |
| ELOVL1 | 7.7 ± 0.6 | 8.6 ± 0.4 | 9.1 ± 0.3* | 7.3 ± 0.3 | 9.1 ± 0.8 | 9.3 ± 0.6* | 9.3 ± 0.4* | 7.3 ± 0.3 |
| FABP5 | 14.0 ± 0.4 | 14.9 ± 0.3* | 14.5 ± 0.2* | 13.5 ± 0.2 | 15.2 ± 0.5* | 15.3 ± 0.4* | 15.0 ± 0.3* | 13.5 ± 0.2 |
| GBA | 9.8 ± 0.5 | 10.8 ± 0.4 | 10.9 ± 0.3* | 9.5 ± 0.3 | 12.8 ± 0.7 | 11.5 ± 0.6* | 11.4 ± 0.5* | 9.5 ± 0.3 |
| ALDH3EA2 | 6.6 ± 1.0 | 9.8 ± 0.8* | 9.2 ± 0.6* | 5.7 ± 0.6 | 8.8 ± 1.1 | 9.2 ± 1.1* | 8.2 ± 0.8 | 5.7 ± 0.6 |
| APOD | 13.7 ± 0.4 | 15.1 ± 0.3* | 15.0 ± 0.2* | 13.0 ± 0.2 | 15.1 ± 0.6* | 14.2 ± 0.5 | 14.3 ± 0.4 | 13.0 ± 0.2 |
| SGPL1 | 7.4 ± 0.5 | 9.0 ± 0.4* | 8.8 ± 0.3* | 7.2 ± 0.3 | 9.0 ± 0.5* | 7.8 ± 0.5 | 8.0 ± 0.3 | 7.2 ± 0.3 |

Cathespins

|  | PT T1 | LPT T1 | FT T1 | Adult | PT T2 | LPT T2 | FT T2 | Adult |
| --- | --- | --- | --- | --- | --- | --- | --- | --- |
| CTSA | 6.8 ± 0.7 | 9.5 ± 0.5 | 9.5 ± 0.4 | 8.2 ± 0.4 | 12.7 ± 0.9* | 11.6 ± 0.8* | 10.6 ± 0.6* | 8.2 ± 0.4 |
| CTSB | 12.6 ± 0.4 | 13.8 ± 0.3* | 12.9 ± 0.2* | 11.9 ± 0.2 | 14.3 ± 0.4* | 13.4 ± 0.4* | 13.6 ± 0.3* | 11.9 ± 0.2 |
| CTSC | 13.8 ± 0.6* | 16.2 ± 0.5* | 15.3* ± 0.4 | 11.0 ± 0.4 | 14.4 ± 0.7* | 14.0 ± 0.6* | 14.2 ± 0.5* | 11.0 ± 0.4 |
