# Supplementary material for: Biomarkers of neonatal skin barrier adaptation reveal substantial differences compared to adult skin
Source: Pediatr Res. 2020 Jun 29;89(5):1208–15. doi: 10.1038/s41390-020-1035-y (PMC8119241; doi:10.1038/s41390-020-1035-y)
Supplement: Supplementary file 4 — Supplementary tableS4 [file 41390_2020_1035_MOESM4_ESM.docx]

**Supplementary Table S4**. Natural Moisturizing Factor. The levels of the components of natural moisturizing factor (NMF) are shown for the three infant groups and adults soon after birth (T1) and 2-3 months later (T2).

|  | **< 34 wks GA (PT)** | **34 - < 37 wks GA (LPT)** | **≥ 37 wks GA (FT)** | **Adults** | **Statistics** |
| --- | --- | --- | --- | --- | --- |
| **Shortly after birth, T1** |  |  |  |  |  |
| Log_10_TotalNMF | 1.87 ± 0.11 | 1.95 ± 0.09 | 1.67 ± 0.07 | 2.39 ± 0.06 | PT, LPT, FT  -S- vs Ad |
| Log_10_PCA | 1.60 ± 0.12 | 1.69 ± 0.09 | 1.42 ± 0.07 | 2.13 ± 0.07 | PT, LPT, FT  -S- vs Ad |
| Log_10_Histidine | 0.91 ± 0.10 | 1.05 ± 0.08 | 0.89 ± 0.06 | 1.71 ± 0.06 | PT, LPT, FT  -S- vs Ad |
| Log_10_CisUCA | Not detected | -0.22 ± 0.18 | 0.23 ± 0.17 | 1.14 ± 0.08 | LPT, FT  -S- vs Ad |
| Log_10_TransUCA | 1.38 ± 0.12 | 1.39 ± 0.09 | 1.16 ± 0.07 | 1.37 ± 0.07 | -NS- |
| Log_10_Proline | 0.57 ± 0.10 | 0.54 ± 0.07 | 0.55 ± 0.06 | 1.10 ± 0.05 | PT, LPT, FT  -S- vs Ad |
| Log_10_TotalUCA | 1.38 ± 0.11 | 1.39 ± 0.09 | 1.18 ± 0.06 | 1.60 ± 0.06 | FT -S- vs Ad |
| Log_10_His/  TotalUCA | -0.47 ± 0.08 | -0.34 ± 0.06 | -0.37 ± 0.05 | 0.11 ± 0.05 | PT, LPT, FT  -S- vs Ad |
| Log_10_CisUCA/  TotalUCA | Not detected | -1.82 ± 0.19 | -1.31 ± 0.18 | -0.49 ± 0.08 | LPT, FT  -S- vs Ad |
| **2-3 months later, T2** |  |  |  |  |  |
| Log_10_TotalNMF | 2.81 ± 0.07 | 2.72 ± 0.06 | 2.72 ± 0.04 | 2.39 ± 0.06 | PT, LPT, FT  -S- vs Ad |
| Log_10_PCA | 2.55 ± 0.07 | 2.45 ± 0.08 | 2.47 ± 0.05 | 2.13 0 ±.04 | PT, LPT, FT  -S- vs Ad |
| Log_10_Histidine | 2.02 ± 0.08 | 1.95 ± 0.07 | 1.86 ± 0.05 | 1.71 ± 0.04 | PT, LPT  -S- vs Ad |
| Log_10_CisUCA | 0.30 ± 0.13 | 0.62 ± 0.14 | 0.68 ± 0.10 | 1.14 ± 0.07 | PT, LPT, FT  -S- vs Ad |
| Log_10_TransUCA | 2.17 ± 0.11 | 2.18 ± 0.10 | 2.08 ± 0.07 | 1.37 ± 0.06 | PT, LPT, FT  -S- vs Ad |
| Log_10_Proline | 1.48 ± 0.07 | 1.48 ± 0.07 | 1.42 ± 0.05 | 1.10 ± 0.04 | PT, LPT, FT  -S- vs Ad |
| Log_10_TotalUCA | 2.18 ± 0.10 | 2.18 ± 0.09 | 2.12 ± 0.06 | 1.60 ± 0.06 | PT, LPT, FT  -S- vs Ad |
| Log_10_His/  TotalUCA | -0.16 ± 0.10 | -0.23 ± 0.09 | -0.26 ± 0.07 | 0.11 ± 0.06 | LPT, FT  -S- vs Ad |
| Log_10_CisUCA/  TotalUCA | -1.91 ± 0.16 | -1.71 ± 0.18 | -1.48 ± 0.12 | -0.49 ± 0.09 | PT, LPT, FT  -S- vs Ad |

*Indicated here are the post-hoc comparisons (Bonferroni) with p < 0.05 from general linear models analyses of the four groups where the model F statistic is significant at p < 0.05. -S- indicates significant pairwise comparisons; -NS- indicates not significant; PT indicates infants < 34 wks GA; LPT indicates 34 - < 37 wks GA; FT indicates full-term (≥ 37 wks GA).
