# Supplementary figures and images for: Biomarkers of neonatal skin barrier adaptation reveal substantial differences compared to adult skin
Source: Pediatr Res. 2020 Jun 29;89(5):1208–15. doi: 10.1038/s41390-020-1035-y (PMC8119241; doi:10.1038/s41390-020-1035-y)

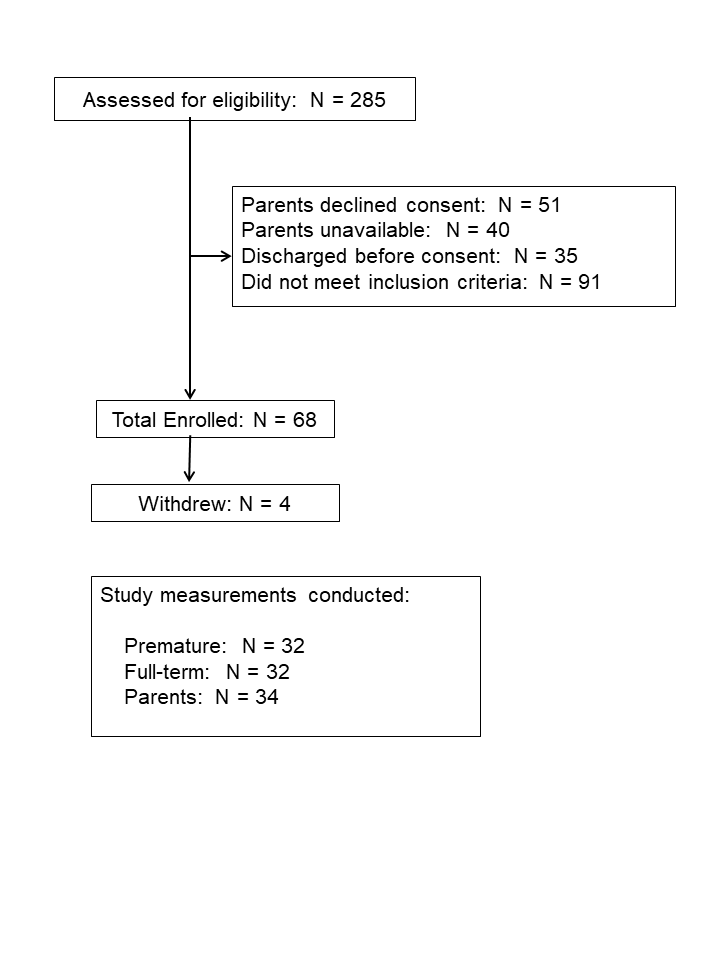

Supplement: Supplementary file 5 — Supplementary figure [file 41390_2020_1035_MOESM5_ESM.tif]
